# TGF-β is insufficient to induce adipocyte state loss without concurrent PPARγ downregulation

**DOI:** 10.1038/s41598-020-71100-z

**Published:** 2020-08-21

**Authors:** Brooks Taylor, Arnav Shah, Ewa Bielczyk-Maczyńska

**Affiliations:** 1 Department of Chemical and Systems Biology, Stanford University, Stanford, CA USA; 2 Present Address: Division of Cardiovascular Medicine, Department of Medicine, Stanford University, Stanford, CA USA

**Keywords:** Single-cell imaging, Time series, Mechanisms of disease, Transdifferentiation

## Abstract

Cell plasticity, the ability of differentiated cells to convert into other cell types, underlies the pathogenesis of many diseases including the transdifferentiation of adipocytes (fat cells) into myofibroblasts in the pathogenesis of dermal fibrosis. Loss of adipocyte identity is an early step in different types of adipocyte plasticity. In this study, we determine the dynamics of adipocyte state loss in response to the profibrotic cytokine TGF-β. We use two complementary approaches, lineage tracing and live fluorescent microscopy, which both allow for robust quantitative tracking of adipocyte identity loss at the single-cell level. We find that the intracellular TGF-β signaling in adipocytes is inhibited by the transcriptional factor PPARγ, specifically by its ubiquitously expressed isoform PPARγ1. However, TGF-β can lead to adipocyte state loss when it is present simultaneously with another stimulus. Our findings establish that an integration of stimuli occurring in a specific order is pivotal for adipocyte state loss which underlies adipocyte plasticity. Our results also suggest the possibility of a more general switch-like mechanism between adipogenic and profibrotic molecular states.

## Introduction

Cell plasticity, the ability of differentiated cells to convert into other cell types, underlies the pathogenesis of many diseases including diabetes^1^ and cancer^2^. Adipocyte (fat cell) plasticity includes reversible dedifferentiation into proliferative adipocyte progenitors in the mammary gland^3^ and skin^4^, as well as a conversion of adipocytes into myofibroblasts. The fate switch to a myofibroblast identity can occur both during skin wound healing^5^ and in the pathogenesis of dermal fibrosis, where it is induced by the profibrotic cytokine TGF-β^6^. Adipocyte plasticity involves the loss of expression of adipocyte marker proteins, suggesting a true cell state switch^4,6^.

While the molecular networks regulating adipocyte differentiation have been relatively well described^7^, the mechanism of adipocyte identity loss remains not understood^6^. PPARγ, a key adipogenic transcription factor which is sufficient and required to drive adipocyte differentiation^8,9^ is known to be critical for maintaining adipocyte functions such as insulin sensitivity^10,11^. However, on its own the loss of PPARγ expression in mature adipocytes is insufficient to cause a reversal to another cell state in an in vitro model^12^. In vivo induced PPARγ loss specifically in adipocytes results in cell death^10,11,13^. The reasons for the discrepancy between the in vitro and in vivo effects of PPARγ loss are not well understood.

One of the main limitations in understanding the molecular mechanisms of adipocyte plasticity is the current lack of appropriate in vitro models capable of reliable identification of a switch from adipocytes into other cell states at the single-cell level, in a manner comparable to the golden standard of lineage tracing used in vivo^6^. The use of bulk methods carries the risk of experimental artifacts resulting from contamination from other cellular sources, such as undifferentiated cells.

Here we present two complementary single-cell approaches which allow for robust quantitative tracking of adipocyte identity loss at the single-cell level. Using these approaches, we investigated the dynamics of adipocyte identity loss following the stimulation with TGF-β, a potent profibrotic cytokine^14^ which was previously linked with adipocyte state loss in a model of dermal fibrosis^6^. We show that high expression of PPARγ in adipocytes is linked with the inhibition of the intracellular TGF-β signaling in the presence of the TGF-β cytokine. However, TGF-β can lead to adipocyte state loss when it is present simultaneously with another stimulus, which appears to be linked with cell pulling or changes in cell adhesion. Together, these findings establish that an integration of stimuli occurring in a specific order is required to drive adipocyte state loss which underlies adipocyte plasticity. Our findings shed light on the discrepancies between in vitro and in vivo studies on adipocyte state loss, and implicate mechanical stimuli as possibly mediating adipocyte plasticity.

## Results

### TGF-β stimulation by itself does not lead to the loss of adipocyte state in vitro

**Figure 1:**
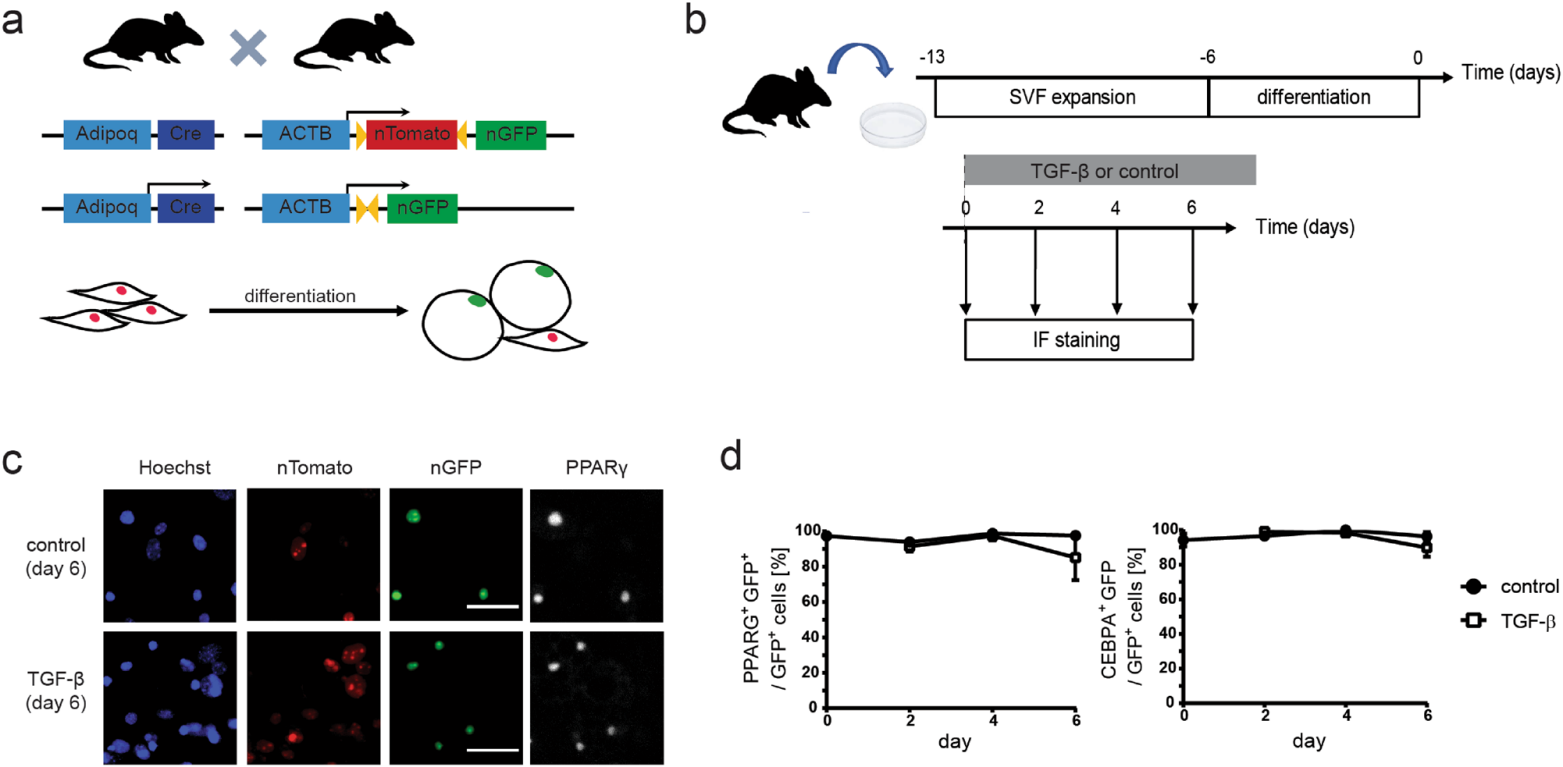
TGF-β stimulation does not induce the loss of adipocyte marker expression under standard culture conditions in primary mouse adipocytes differentiated ex vivo. (**a**) Schematic of the transgenic mouse model used. (**b**) Experiment outline to test the effect of TGF-β on primary adipocytes using immunofluorescent detection of GFP and adipocyte markers PPARγ and C/EBPα. Primary SVF cells from *Adipoq:Cre nT/nG* mice were expanded and differentiated into adipocytes in vitro. TGF-β was added to the culture media at the end of differentiation (day 0) and cells were analyzed at days 0, 2, 4 and 6 using immunofluorescent staining. (**c**) Representative fluorescent images of staining against PPARγ at day 6 after adding stimulus. GFP expression is colocalized with PPARγ expression in the nuclei of both control and TGF-β-treated cells. Scale bar: 50 µm. (**d**) Percentage of GFP-positive cells expressing adipocyte markers PPARγ and C/EBPα. Two-tailed Student *t* tests with Benjamini–Hochberg correction; FDR = 0.01; n = 3–8 technical replicates, all time points *p* > 0.05.

First, in order to quantitatively study adipocyte state loss we adapted the lineage tracing, a technique widely used for determining that a particular cell belongs to adipocyte lineage in vivo^15^, for in vitro use. In short, we used cells derived from either of two transgenic mouse models: *Adipoq:Cre mT/mG* and *Adipoq:Cre nT/nG*. In both these models the adipocyte-specific adiponectin promoter drives the expression of Cre recombinase, which causes an irreversible switch from red (Tomato) to green (GFP) fluorescence specifically in adipocytes (Fig. 1a). Adiponectin has been shown to be the most reliable marker for lineage tracing of mature adipocytes because its expression does not label adipose progenitor cells or other non-adipogenic populations present in the fat pad^15^. Fluorescent protein expression can be either localized to cell membrane (in the *Adipoq:Cre mT/mG* model) or to the nucleus (in the *Adipoq:Cre nT/nG* model), allowing to detect cells derived from adipocytes under various conditions, such as varying levels of cell confluence. First, to obtain primary adipocytes, we followed established protocols^16^ to isolate the preadipocyte-containing stromal vascular fraction (SVF) from subcutaneous inguinal fat pads of *Adipoq:Cre mT/mG* and *Adipoq:Cre nT/nG* mice and subjected it to an adipogenic differentiation protocol ex vivo. Over time we observed the expected switch in fluorescence from red (Tomato) to green (GFP) in a fraction of SVF cells. Furthermore, the GFP-positive cells were characterized by the co-expression of adipocyte markers PPARγ and C\EBPα, confirming that the GFP-positive cells were adipocytes (Supplementary Fig. S1 online). At the end of the differentiation protocol cells were subjected to TGF-β treatment for up to six days and analyzed for GFP, PPARγ and C\EBPα expression using immunofluorescent staining (Fig. 1b). To our surprise, virtually all GFP-positive cells maintained high expression of adipocyte markers PPARγ and C\EBPα throughout six days of analysis, irrespective of TGF-β treatment (Fig. 1c,d), suggesting that TGF-β does not induce adipocyte plasticity in this cell model under standard conditions, contrary to previous reports using differentiated human adipose tissue-derived progenitor cells (ADSCs)^6^. Of note, we observed progressive decrease in the total number of GFP-positive cells under TGF-β treatment but not in control conditions, suggesting adipocyte loss due to TGF-β-induced apoptosis (Supplementary Fig. S2 online).

**Figure 2:**
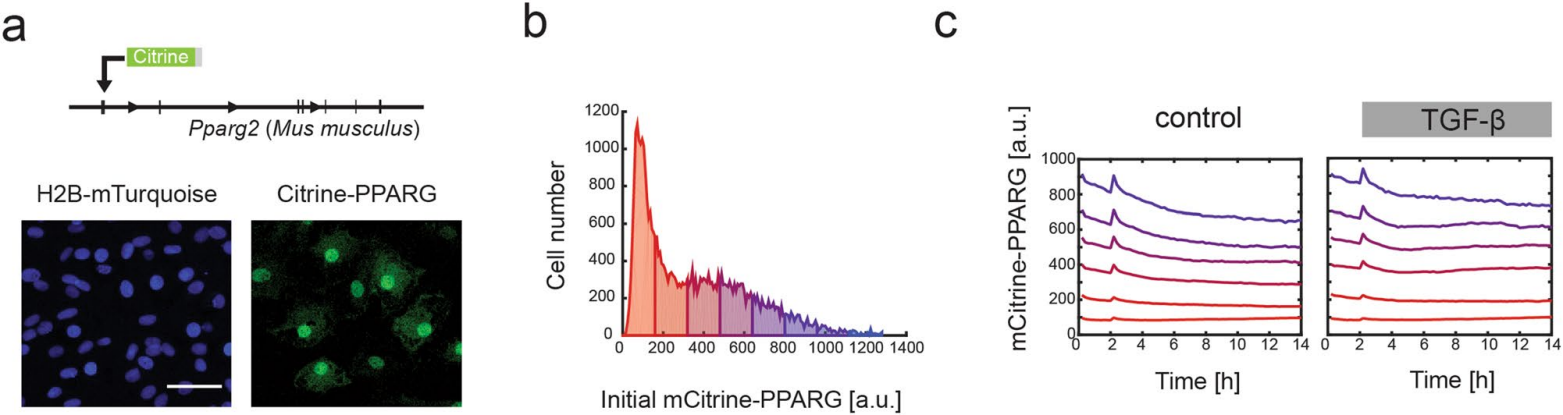
The mCitrine-PPARG cell line can be used to track the endogenous expression of the adipogenic marker PPARγ2 at the single-cell level. (**a**) Schematic and representative fluorescent images of the mCitrine-PPARG OP9 cell line which also includes a fluorescent nuclear marker H2B-mTurquoise (CFP). Scale bar: 100 um. (**b**) Distribution of single-cell mCitrine-PPARγ expression at the end of differentiation protocol (day 4). Cells were binned based on the nuclear mCitrine signal and the bins were color-coded. (**c**) Time course analysis of median mCitrine expression in six bins depending on initial mCitrine expression in differentiated non-replated cells. Data for untreated control and cells treated with 2 ng/ml TGF-β added at 2 h are shown.

In order to validate whether TGF-β does not lead to adipocyte state loss, we decided to use another adipocyte in vitro cell model which allows for tracking of fate of individual adipocytes. To this end, we employed the mouse adipogenic OP9 cell line which was previously engineered to include a fluorescent tag mCitrine on the endogenous PPARγ2 protein (mCitrine-PPARG line). This cell line allows us to precisely measure the expression dynamics of the adipocyte marker PPARγ2 using live fluorescence microscopy and quantification of mCitrine expression in thousands of cells while they are subjected to different perturbations^17^ (Fig. 2a). At the end of a standard four-day differentiation protocol, mCitrine-PPARG cells are characterized by a bimodal distribution of individual mCitrine expression levels (Fig. 2b), with high expression of mCitrine-PPARγ in differentiated adipocytes and markedly lower expression in undifferentiated preadipocytes^17^. When TGF-β was applied to a population of mixed differentiated and undifferentiated mCitrine-PPARG cells obtained at the end of the differentiation protocol, we have not observed a decrease in the mCitrine signal in any of the adipocyte groups of cells within 12 h after TGF-β addition, supporting that TGF-β has no effect on adipocyte cell state in this system (Fig. 2c, additional tracking for 36 h in Supplementary Fig. S3 online). Altogether, our observations using both primary mouse adipocytes and the mCitrine-PPARG OP9 cells support the view that under standard in vitro tissue culture conditions TGF-β is insufficient to induce the loss of adipocyte state.

### Downregulation of PPARγ in OP9 adipocytes is associated with TGF-β-induced cell cluster formation

**Figure 3:**
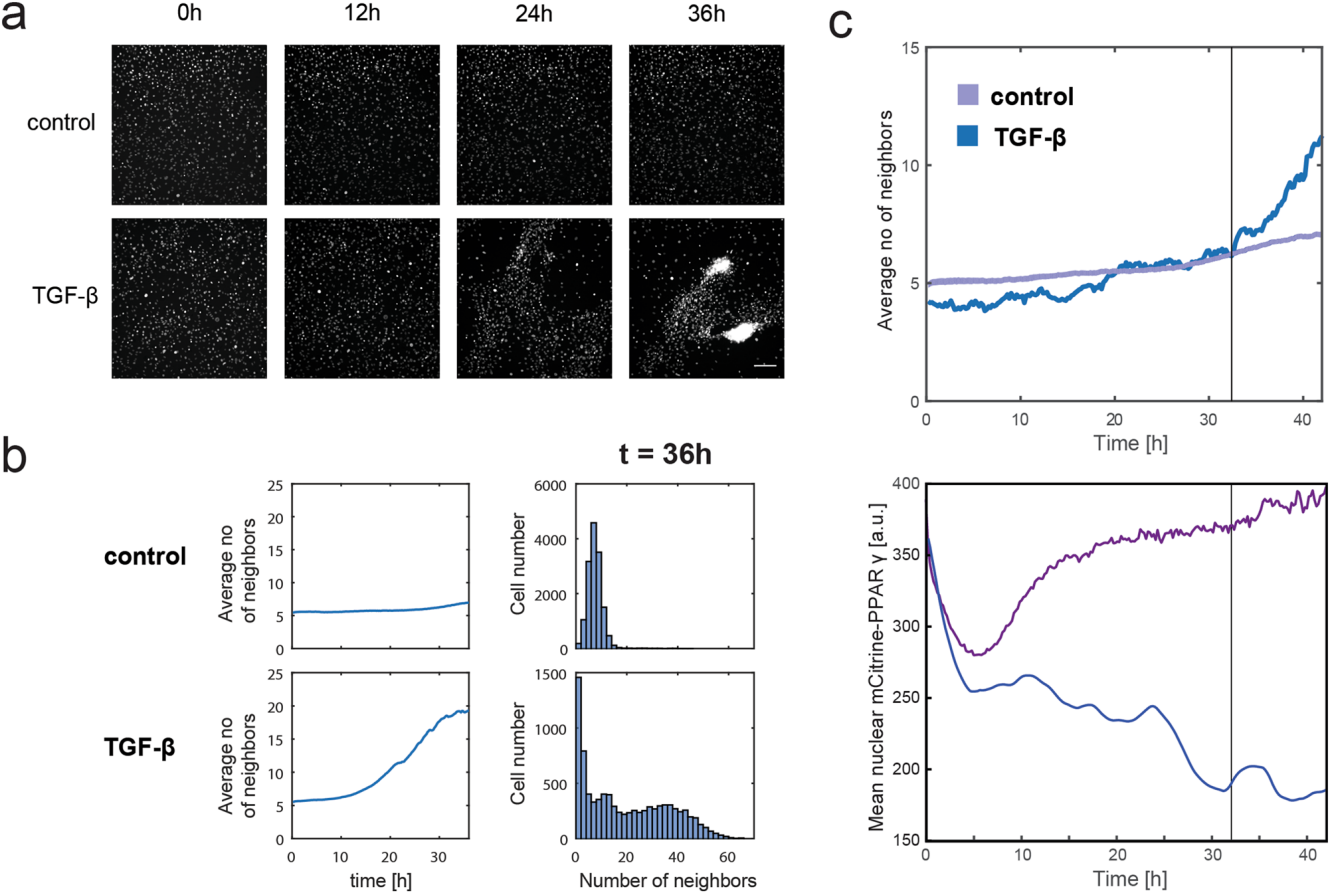
Downregulation of adipocyte marker expression in mCitrine-PPARG cells undergoing TGF-β-induced cell cluster formation. (**a**) Representative fluorescent images of mCitrine-PPARG OP9 cells in the CFP channel to visualize the nuclei of cells forming clumps in a TGF-β-dependent manner in individual control and TGF-β-treated sites. TGF-β at 2 ng/ml was applied at 0 h. Scale bar: 200 µm. (**b**) Quantification of the clumping phenomenon using the analysis of average number of neighbor nuclei, quantified as other nuclei present within 38 µm radius of each cell’s nucleus. Average number of neighbors plotted for the whole 36 h of analysis and single-cell distribution of neighbor number at 36 h are shown. (**c**) Representative single-cell mCitrine-PPARG expression level in a PPARγ-high adipocyte. Upper plot: Average number of neighbors in control (purple, average ± S.E.M., n = 6 technical replicates) and in a single TGF-β-treated well (blue) during 42 h-long live experiment. Lower plot: representative time course of mCitrine-PPARG expression in an individual cell from the same TGF-β-treated well (blue) and from adipocyte bin with a comparable initial mCitrine-PPARG level in control conditions (purple). Vertical lines denotes the time point when average number of neighbors after TGF-β application exceeds average number of neighbors in control, indicating the beginning of clumping.

Thanks to the application of lineage tracing we were able to successfully track the single-cell expression of adipocyte markers PPARγ and C\EBPα in primary adipocytes over six days following TGF-β treatment (Fig. 1d). However, in the mCitrine-PPARG OP9 cell model the incubation of cells with TGF-β for over 36 h invariably led to the formation of distinct cell clusters, which severely limited our single-cell tracking capacity (Fig. 3a,b, Supplementary Fig. S3 online). The clusters closely resembled the phenomenon previously described in the adipogenic cell line 3T3-L1 following treatment with the Wnt3a cytokine^18^. Strikingly, Wnt3a-induced cell clusters were reported to co-occur with cell cycle re-entry of lipid-filled 3T3-L1 adipocytes, indicative of adipocyte plasticity^18^. We hypothesized that the appearance of cell clusters under TGF-β stimulation may also be linked with adipocyte plasticity. Due to the difficulties in single-cell tracking associated with cell cluster formation, only very few mCitrine-PPARG-positive adipocytes which did not contribute to cell clusters could be reliably followed over prolonged periods of time. However, analysis of these cells revealed a progressive loss of mCitrine expression, which appeared to begin prior to noticeable cluster formation (Fig. 3c). The contradiction between the early PPARγ downregulation in some adipocytes treated with TGF-β and the apparent lack of general PPARγ downregulation in TGF-β-treated adipocytes prior to clumping (Fig. 2) suggests that the minority of adipocytes which undergo plasticity in response to TGF-β may be enriched in the population which is trackable.

**Figure 4:**
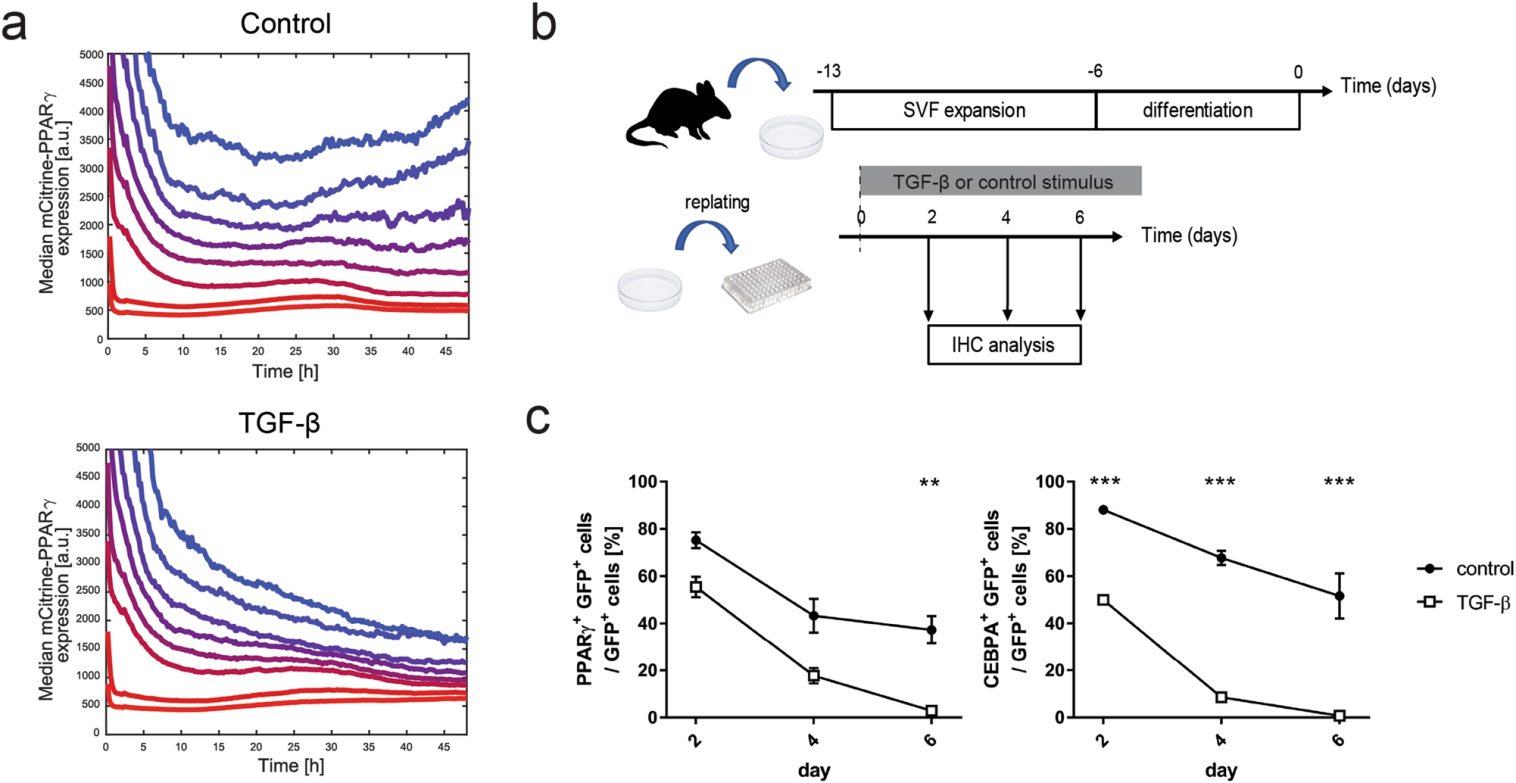
Replating sensitizes adipocytes to TGF-β-induced loss of adipocyte marker expression. (**a**) Time course analysis of median mCitrine expression in differentiated mCitrine-PPARG OP9 cells subjected to replating at 0 h. All cells were grouped into eight bins depending on the initial mCitrine expression. Cells were either treated with 2 ng/ml TGF-β added at the time of replating or not. Median mCitrine expression for each bin is shown. (**b**) Outline of the experiment to test the effect of cell replating on TGF-β-induced loss of adipocyte marker expression in primary mouse adipocytes differentiated ex vivo. (**c**) The dynamics of TGF-β-induced loss of adipocyte marker expression in SVF-derived primary adipocytes. Percentage of GFP-positive cells which expressed adipocyte markers PPARγ and C/EBPα at different time points following replating. n = 4 technical replicates, GFP-positive cells/replicate/time point > 32. Average and S.E.M. shown, two-tailed Student *t* tests with Benjamini–Hochberg correction; FDR = 0.01; **p < 0.01; ***p < 0.001.

We hypothesized that mechanical forces inflicted by other cells, changes in cellular adhesion and/or decrease in local confluence may be affecting adipocytes before and during cell cluster formation and contribute to the loss of mCitrine-PPARG expression, either directly or indirectly. In order to test this hypothesis, we replated differentiated mCitrine-PPARG OP9 cells at subconfluence and simultaneously subjected them to TGF-β stimulation. As expected, TGF-β caused a significant downregulation of mCitrine in adipocytes compared to control adipocytes which had only been replated but not treated with TGF-β (Fig. 4a). Similarly, we observed downregulation of adipocyte marker expression in *Adipoq:Cre mT/mG* adipocytes treated with TGF-β when they were replated at subconfluence at the end of differentiation (Fig. 4b,c), in stark contrast to our earlier observations of non-replated primary adipocytes treated with TGF-β (Fig. 1). Altogether, this set of experiments suggested that adipocytes are not permanently locked in their high-PPARγ state but TGF-β stimulation by itself is insufficient to cause adipocyte plasticity.

### TGF-β signaling is inhibited in adipocytes

TGF-β mediates its biological effects through the cell membrane TGF-β receptor. Once activated by the binding of the TGF-β cytokine, the receptor phosphorylates the effector protein SMAD2 or SMAD3 (SMAD2/3). Activated SMAD2/3, together with SMAD4, translocate to cell nucleus to stimulate TGF-β-dependent transcription^19^.

**Figure 5:**
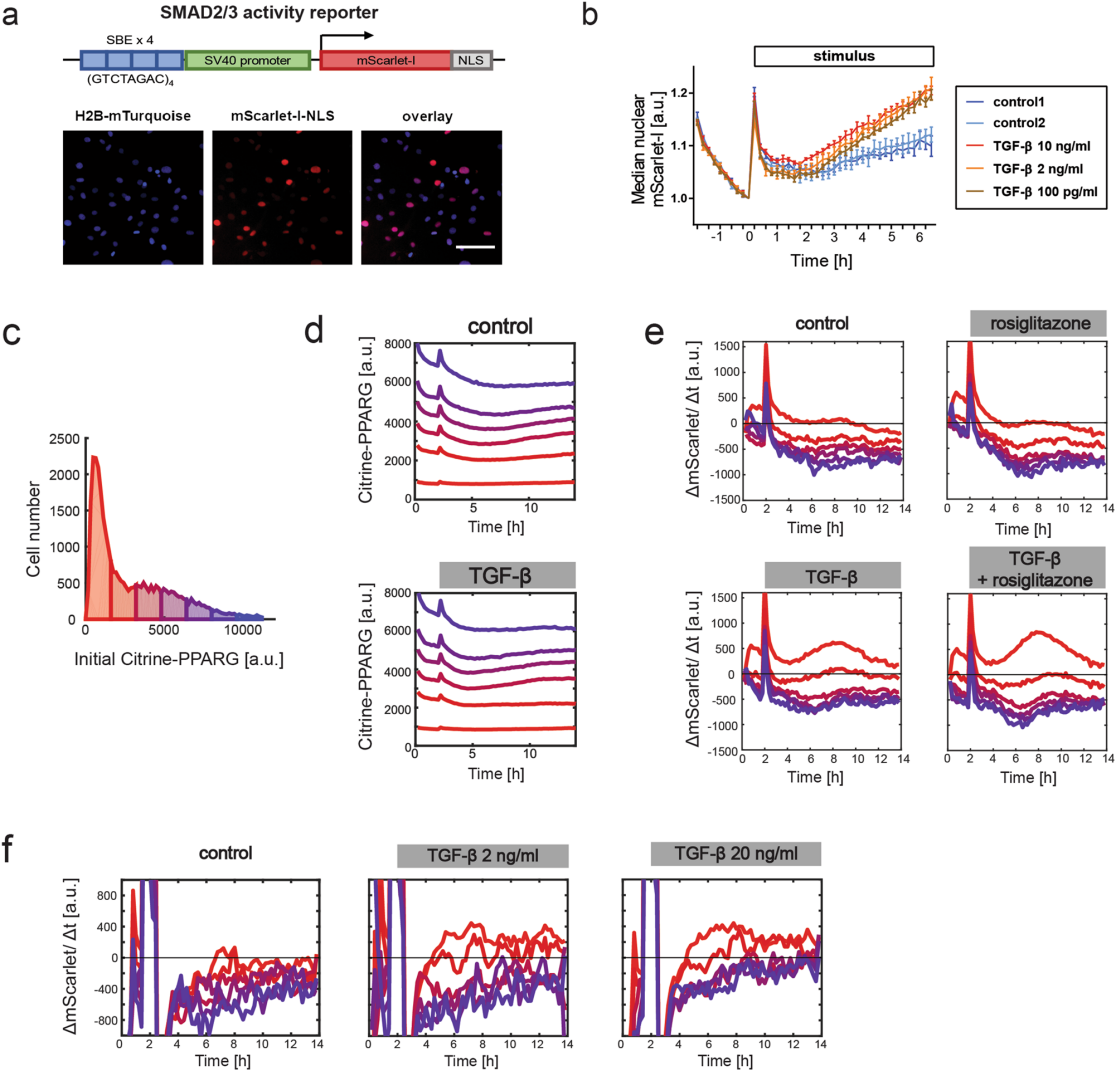
TGF-β signaling activation is restricted to PPARγ-low cells in a population of differentiated and undifferentiated mCitrine-PPARG OP9 cells. (**a**) Schematic of the live fluorescent reporter of SMAD2/3 transcriptional response, SBE4:mScarlet-I-NLS. Representative fluorescent images of undifferentiated cells are shown. Scale bar: 100 µm. (**b**) The reporter allows detection of TGF-β signaling pathway activity. Undifferentiated SBE4:mScarlet-I-NLS OP9 cells were treated with various concentrations of TGF-β after initial 2 h of pre-incubation with basal media. For each single cell trace, nuclear signal was normalized by t = 0 h. Mean from n = 3 technical replicates and S.E.M. are shown. (**c**–**e**) TGF-β applied at 2 h. Results of one experiment representative for three independent experiments. (**c**) The distribution of single-cell mCitrine expression in the last frame before stimulus addition (2 h), used to assign cells to bins in panels (**d**,**e**), shown for the control group. (**d**) Time course analysis of median mCitrine expression in six bins depending on initial mCitrine expression in differentiated non-replated mCitrine-PPARG SBE4:mScarlet-I-NLS OP9 cells. (**e**) Strong reporter upregulation, indicated by positive values of the change in integrated nuclear mScarlet-I signal over time (ΔmScarlet/Δt), in the cell bin with the lowest initial mCitrine expression. Median trace for each bin is shown. 12 h of treatment with TGF-β (2 ng/ml), rosiglitazone (1 µM), TGF-β and rosiglitazone, or with basal media in control, beginning after 2 h of pre-incubation with basal media. Median mCitrine expression traces for each bin are shown. (**f**) Increasing TGF-β dose tenfold does not lead to the upregulation of SBE4:mScarlet-I-NLS reporter in mCitrine-low cell populations. Consistent changes in fluorescence during the first 2 h of experiment are attributable to illumination settings.

PPARγ can inhibit SMAD3^20,21^ and in turn SMAD3 is inhibitory to the PPARγ positive feedback partner C\EBPα^22,23^, which led us to hypothesize the existence of a double-negative feedback system between TGF-β signaling and the adipocyte transcriptional network. To test this hypothesis, we constructed a live fluorescent reporter of SMAD2/3 transcriptional response (SBE4:mScarlet-I-NLS, Fig. 5a) and introduced it into the mCitrine-PPARG OP9 cell line. This reporter enables detection of rapid changes in TGF-β signaling-dependent gene expression (Fig. 5b). When a population of differentiated mCitrine-PPARG OP9 cells was treated with TGF-β, the activation of the TGF-β reporter was restricted to cells with the lowest initial mCitrine expression, and co-treatment with the PPARγ agonist rosiglitazone did not lower TGF-β signaling activity in this group (Fig. 5c–e). The lack of activation of the TGF-β reporter in mCitrine-PPARG-high cells was not due to low TGF-β concentration as increasing the TGF-β dose tenfold did not cause TGF-β reporter upregulation in cells with high mCitrine expression levels (Fig. 5f). We concluded that canonical TGF-β signaling is inhibited in adipocytes.

**Figure 6:**
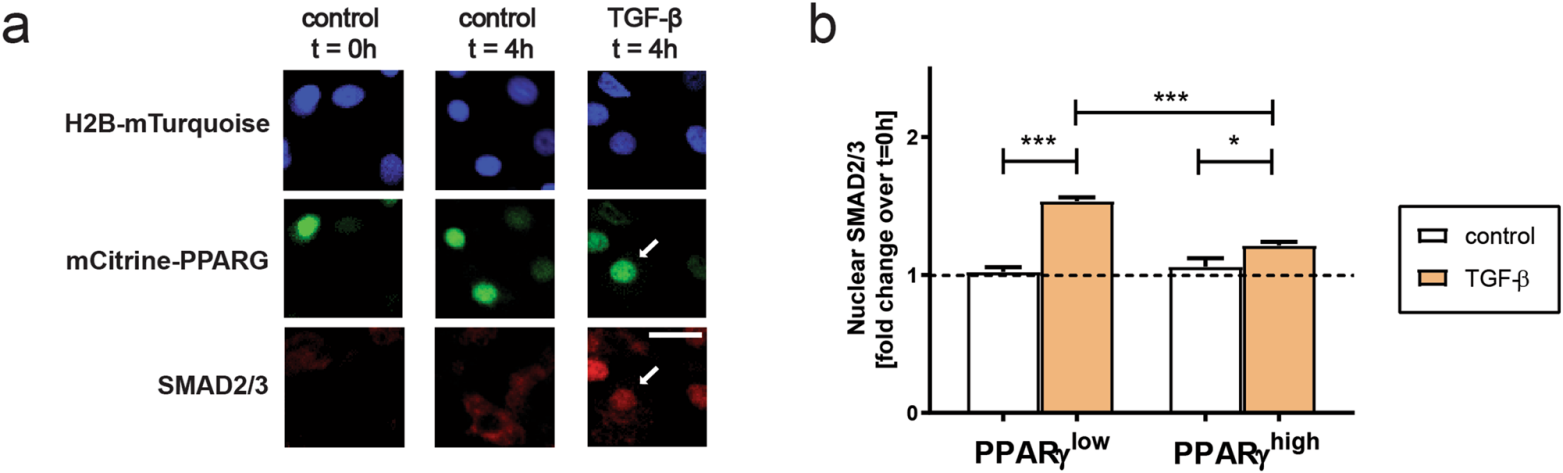
The block in TGF-β signaling transduction in adipocytes is at least partially due to a block in SMAD2/3 translocation to the nucleus. (**a**) TGF-β treatment leads to SMAD2/3 translocation into the nucleus within 4 h both in PPARγ-low and PPARγ-high cells. Differentiated non-replated mCitrine-PPARG OP9 cells were treated with TGF-β and subjected to immunoflurescent staining of SMAD2/3 and PPARγ. Arrows denote a PPARγ-high cell with SMAD2/3 localizing to the nucleus after 4 h of TGF-β treatment. Scale bar: 100 µm. (**b**) Quantification of nuclear SMAD2/3 intensity in PPARγ-high and PPARγ-low cells treated for 4 h with TGF-β or control media, normalized to values at 0 h. Ordinary one-way ANOVA with Sidak’s multiple comparison test; *p < 0.05; ***p < 0.001.

Next, we decided to test whether in adipocytes there is an indication of TGF-β signaling inhibition at earlier steps of the TGF-β signaling cascade than the transcriptional response. To this end, we tested whether the translocation of SMAD2/3 from cytoplasm to the nucleus in response to TGF-β is affected (Fig. 6). We observed SMAD2/3 translocation to the nucleus irrespective of PPARγ expression levels, even though the nuclear translocation of SMAD2/3 under TGF-β was significantly weaker in PPARγ-high compared to PPARγ-low cells (Fig. 6b). We concluded that SMAD2/3 translocation to the nucleus is inhibited in PPARγ-expressing cells. However, it is likely that another block in signaling occurs at a later step in the signaling cascade, perhaps through affecting transcriptional activity of SMAD2/3. Interestingly, prolonged stimulation of mCitrine-PPARG SBE4:mScarlet-I-NLS cells with TGF-β revealed that eventual activation of TGF-β signaling in adipocytes temporally followed cell clump formation (Supplementary Fig. S5 online), supporting the idea that TGF-β can cause a loss of adipocyte state but only if another stimulus is present.

### PPARγ inhibits TGF-β signaling

**Figure 7:**
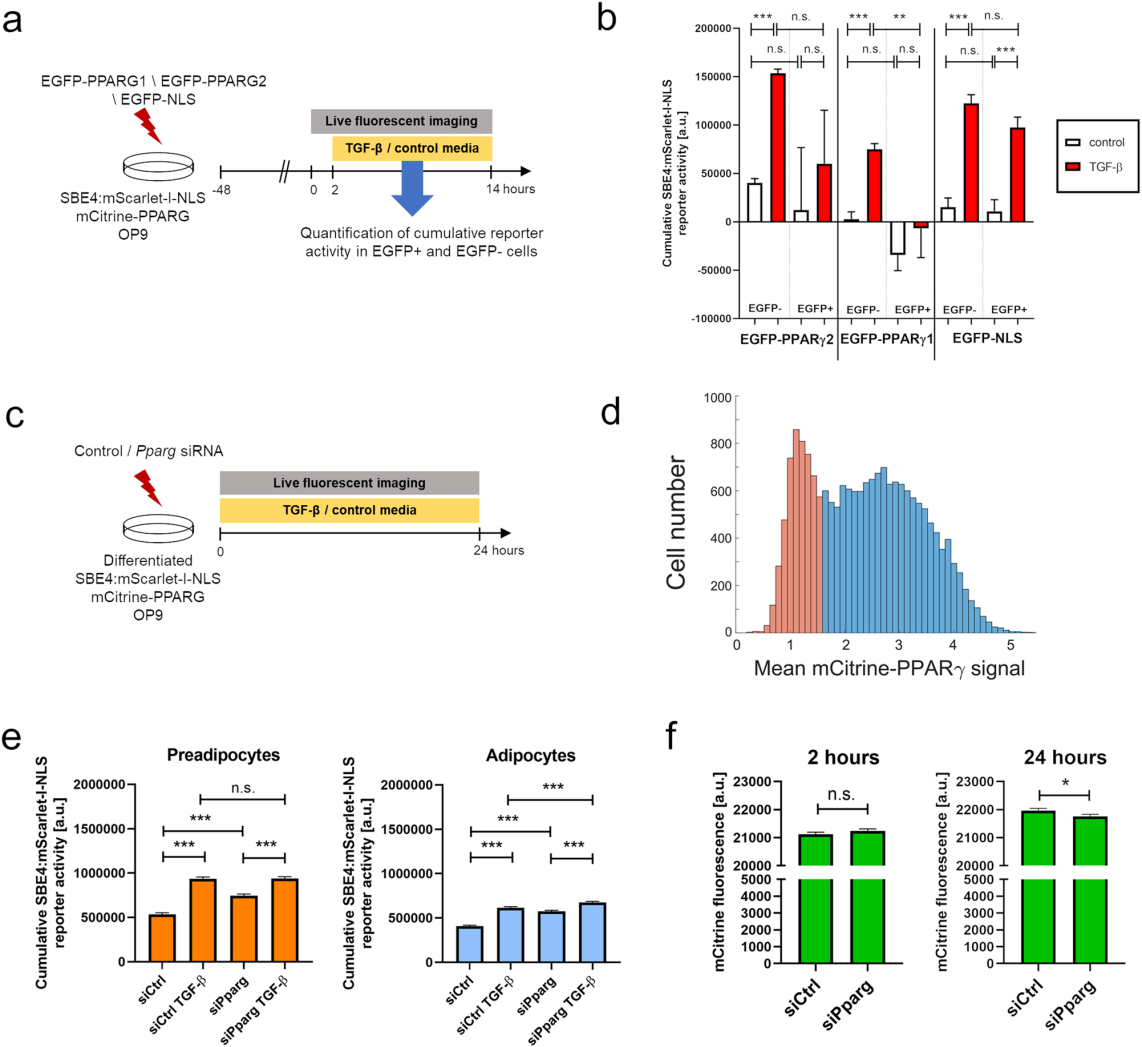
PPARγ inhibits TGF-β signaling in mCitrine-PPARG SBE4:mScarlet-I-NLS OP9 cells. (**a**) Outline of the method used to quantify TGF-β signaling activation depending on the overexpression of custom constructs. To prevent basal differentiation, cells were kept below 50% of confluence throughout the experiment. (**b**) Quantification of cumulative SBE4:mScarlet-I-NLS activity at the single-cell level during 12 h after TGF-β stimulation in transfected (EGFP +) and control untransfected (EGFP-) cells in the same wells. Results of one experiment representative for three independent experiments. (**c**) Outline of the method used to quantify TGF-β signaling activity depending on *Pparg* knock-down. SBE4:mScarlet-I-NLS mCitrine-PPARG cells were differentiated, followed by transfection with either *Pparg* siRNA or control non-targeting siRNA. (**d**) mCitrine-PPARG expression at the beginning of imaging was used to classify cells as either preadipocytes (orange) or adipocytes (blue). (**e**) Quantification of cumulative SBE4:mScarlet-I-NLS activity at the single-cell level during 24 h after siRNA transfection in preadipocytes and adipocytes. Ordinary one-way ANOVA with Sidak’s multiple comparisons test. (**f**) Determination of siRNA efficiency by the quantification of mCitrine-PPARG expression at 2 h and 24 h in all cells treated with *Pparg* siRNA or control non-targeting siRNA. Two-tailed Student *t*-tests. **p < 0.01; ***p < 0.001, n.s.—not significant. Average + S.E.M. shown.

We next sought to determine if PPARγ is in fact causative for the observed block in TGF-β signaling in adipocytes, or whether its expression is merely correlated with another causative molecular mechanism which is also present in differentiated adipocytes. PPARγ has two major isoforms: PPARγ1, expressed in several cell types^24^, and adipocyte-specific PPARγ2^8^. To establish whether PPARγ causes the block of TGF-β signaling transduction, we used transient overexpression of human PPARγ1 and PPARγ2 isoforms fused to a fluorescent protein EGFP, and overexpression of EGFP fused with a nuclear localization sequence (NLS) served as a control. First, we verified that transient overexpression of either construct in non-confluent undifferentiated OP9 cells did not induce upregulation of endogenous PPARγ (Supplementary Fig. S6 online). Next, we introduced the overexpression constructs into undifferentiated non-confluent SBE4:mScarlet-I-NLS mCitrine-PPARG OP9 cells and subsequently quantified TGF-β signaling activation in response to TGF-β stimulation at the single cell level (Fig. 7a). Through the comparison of TGF-β signaling response in transfected cells (EGFP+) and untransfected control cells (EGFP−) present in the same wells, we observed significantly lower activity of the reporter following EGFP-PPARγ1 but not EGFP-PPARγ2 nor EGFP-NLS overexpression (Fig. 7b). Both plasmids encoding PPARγ showed a markedly lower transfection efficiency compared to the control construct (Supplementary Table S1 online), which may indicate a negative effect of PPARγ overexpression on cell survival.

Conversely, to determine if PPARγ downregulation is sufficient to increase TGF-β signaling activity we silenced *Pparg* expression through siRNA-mediated gene knock-down of *Pparg* in differentiated SBE4:mScarlet-I-NLS mCitrine-PPARG OP9 cells (Fig. 7c). Quantification of TGF-β reporter activity over 24 h following siRNA transfection revealed that *Pparg* knock-down led to a significant increase of TGF-β signaling in adipocytes and preadipocytes both under control conditions and when TGF-β was present in the culture media, with the exception of TGF-β treated-preadipocytes where *Pparg* knock-down did not cause a further increase in reporter activity (Fig. 7d,e). It is possible that TGF-β-treated preadipocytes display a maximal level of the TGF-β reporter activation which prohibits a further increase by *Pparg* knock-down. The level of knock-down of PPARγ at the protein level was low, although statistically significant (Fig. 7f). Cumulatively, the overexpression and knock-down experiments indicated that TGF-β signaling is negatively regulated by PPARγ.

### Temporal coexistence of stimuli is required for adipocyte state loss

**Figure 8:**
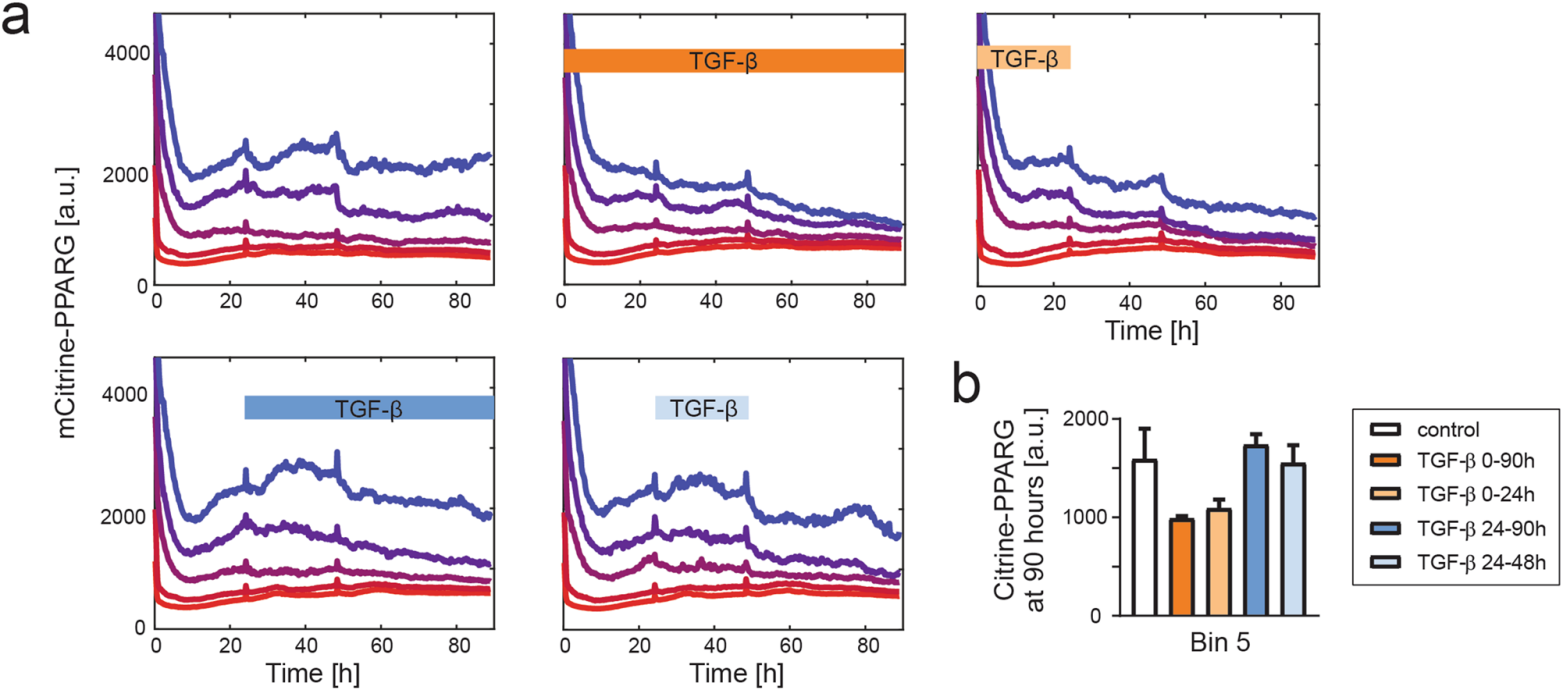
PPARγ downregulation requires temporal coexistence of TGF-β stimulation and another stimulus. (**a**) Single-cell analysis of mCitrine expression in replated mCitrine-PPARG OP9 cells at the end of differentiation protocol. Cells were grouped into 5 bins depending on the mCitrine expression level immediately after replating. Long-term mCitrine expression downregulation in mCitrine-high cells is observed only if TGF-β stimulation occurs during the first 24 h when cells are adhering after replating. Windows of treatment with 2 ng/ml TGF-β are shown. Plots show median traces for each bin. Culture media was replaced at 24 h and 48 h for all conditions. (**b**) Quantification of median endpoint (90 h) mCitrine expression in Bin 5 (the bin with the highest initial mCitrine). n = 4 technical replicates, all with *p* > 0.05.

To obtain deeper understanding of the dynamic interplay between TGF-β and additional stimuli in the loss of adipocyte state, we aimed to alter the timing between the stimuli. To this end, differentiated mCitrine-PPARG OP9 cells were passaged at subconfluence and tracked for 90 h using live-cell fluorescent imaging. The cells were additionally stimulated with TGF-β which was added to the culture media during various time windows (Fig. 8a). Within cells with the highest initial mCitrine expression, we observed a trend towards mCitrine downregulation under TGF-β, but only if the cytokine was applied at the time of cell passaging (TGF-β treatment 0–24 h and 0–90 h). In contrast, TGF-β application beginning 24 h after replating (TGF-β treatment 24–90 h and 24–48 h) did not lead to a noticeable mCitrine downregulation at 90 h compared to the control condition, in which cells were replated but not treated with TGF-β (Fig. 8b). We concluded that long-term downregulation of adipocyte marker expression occurs only when TGF-β stimulation co-occurs with an additional, yet unspecified, stimulus.

Finally, to shed light on the molecular basis of the additional stimulus required for the TGF-β-induced adipocyte state loss in the mCitrine-PPARG OP9 model, we took advantage of an apparent transient drop in mCitrine expression in adipocytes immediately after cells were passaged (Supplementary Fig. S7a online). We reasoned that if this transient disruption of PPARγ expression was caused by a stimulus introduced by passaging, then inhibition of the causative signaling pathway would prevent the mCitrine-PPARG downregulation. To this end, we tested a panel of inhibitors targeting various mechanotransduction pathways by applying them at the time of replating and quantified mCitrine levels at 0, 12 and 24 h after passaging in the subset of cells with the highest initial mCitrine levels. Rho inhibitor I C3 led to a statistically significant increase of mCitrine expression both at 12 h and 24 h while focal adhesion kinase (FAK) and Rho-associated protein kinase (ROCK) inhibitors (PND-1186 and Y27632, respectively) showed no effect (Supplementary Fig. S7b online). We concluded that Rho signaling is a likely candidate for follow-up studies into molecular mechanism of adipocyte state loss as it seems to mediate PPARγ downregulation caused by adipocyte replating.

## Discussion

Loss of the differentiated cell state caused by cell plasticity can result in the inability of the tissue to perform its functions^1^. Therefore, it seems likely that there would be mechanisms in place to prevent the loss of differentiated state in response to transient signals. TGF-β is a widespread cytokine which is synthesized as a latent precursor stored extracellularly and activated by proteins associated with wound healing processes^14^. In addition, TGF-β levels are elevated in obese adipose tissue^25^. Therefore, adipocytes likely are periodically subjected to biologically active TGF-β. Although the presence of TGF-β receptors in the cell membrane is strongly downregulated during adipogenesis^26^, we observed the presence of SMAD2/3 translocation to the nucleus in adipocytes treated with TGF-β, indicating that the transduction of TGF-β signaling in adipocytes is not completely blocked at the receptor level. However, SMAD2/3 translocation to the nucleus was significantly weaker in PPARγ-high cells than in PPARγ-low cells, suggesting that TGF-β signaling is at least partially inhibited in adipocytes at an early step of the TGF-β signaling cascade.

Our observations indicate a key role of the adipogenic transcription factor PPARγ in blocking TGF-β signaling, and therefore, in inhibiting adipocyte state loss. Based on our observation that TGF-β signaling was inhibited both in PPARγ-high cells at the end of a differentiation protocol and in undifferentiated cells in which PPARγ was overexpressed, it seems that at least a temporary drop in PPARγ expression in adipocytes is required to allow the TGF-β signaling to be successfully transduced. Once the TGF-β signal is transduced, however, it can cause PPARγ downregulation, eventually leading to a switch to a non-adipocyte PPARγ-low state.

Interestingly, although the role of PPARγ in the maintenance of the adipocyte state has been addressed before, the results were contradictory and dependent on the experimental system used. In vitro PPARγ downregulation in adipocytes does not lead to major changes in cellular morphology^27^, unless coupled with the overexpression of the potent transcription factor GATA2^12^. On the other hand, PPARγ ablation in vivo leads to the loss of adipocytes, which was interpreted as the result of cell death^10,11,13^. In light of our observations we hypothesize that certain stimuli affecting adipocytes in vivo but not in vitro, such as mechanical forces affecting adipocytes in the context of adipose tissue, may underlie the contradictory effects of PPARγ loss in different experimental systems.

Plasticity may represent one of several processes which can occur in adipocytes stimulated with TGF-β. In our study TGF-β treatment of nonreplated primary cells led to adipocyte loss over time, which suggests that TGF-β signaling in adipocytes may drive apoptosis. In addition, when nonreplated mCitrine-PPARG adipocytes were stimulated with TGF-β, trackable adipocytes undergoing PPARγ downregulation, indicative of plasticity, were rare. However, based on the data on the transcriptional activity of TGF-β signaling in adipocytes, it seems possible that the majority of adipocytes do not transduce TGF-β signaling without being stimulated with an additional PPARγ-disrupting stimulus. The propensity of adipocytes for plasticity and apoptosis under TGF-β stimulation and other plasticity-inducing stimuli remains to be determined.

The ability of the PPARγ1 isoform to inhibit TGF-β-dependent transcriptional response implicates that the PPARγ-mediated block of TGF-β signaling may apply not only to adipocytes, but also to a variety of other cell types which express PPARγ1. Fibrosis, a disease largely driven by TGF-β, affects a variety of tissues. The development and progression of fibrosis is stimulated by myofibroblasts, a cell type which normally arises transiently during wound healing but in fibrosis becomes permanently present in the affected tissue^14,28^. Interestingly, certain tissue-specific cell types which constitute the myofibroblast source in fibrosis, such as hepatic stellate cells in liver^29^ and lipogenic fibroblasts in lung^30^, share molecular characteristics with adipocytes, including PPARγ expression^30,31^. Moreover, PPARγ agonists thiazolidinediones (TZDs) show antifibrotic effects in various tissues^20,32^. It is intriguing that the proadipogenic and profibrotic molecular states may be operated through a molecular switch through the interactions between PPARγ and TGF-β signaling. It is also possible that the principles of the PPARγ-mediated block of TGF-β signaling may apply to various types of fibrotic conversion of cell state, although different levels of PPARγ expression in various cell types could lead to different strength of the block on TGF-β signaling and, as a consequence, to divergent biological responses to identical stimuli. The effects of PPARγ expression on TGF-β signaling in different cell types remain to be investigated.

Our observations on the requirement for signal convergence contrast with the reported robust transdifferentiation of adipocytes into myofibroblasts in response to TGF-β in human adipocytes differentiated from subcutaneous ADSCs^6^. While we cannot exclude that major differences in regulation of adipocyte plasticity exist between murine and human adipose tissue models, the adipocyte-to-myofibroblast switch in the human ADSC-derived adipocyte model remains to be reliably tracked at the single-cell level to exclude the possibility of differentiation of myofibroblasts from other cells besides adipocytes. In addition, while TGF-β-treated differentiated ADSCs did not appear to form cell clusters, that does not exclude the presence of other signals, such as mechanical ones, acting at the cellular level without affecting the general morphology of cells. Future research will be needed to establish whether the TGF-β signaling blockade mediated by PPARγ is relevant to the maintenance of adipocyte state in human adipocytes.

Given our observation that an undefined additional stimulus needs to co-exist with TGF-β stimulation to cause the loss of adipocyte state, in vitro 2D models do not seem well suited for addressing the question of the molecular identity of the additional stimulus required. In fact, certain stimuli associated with replating, such as the loss of cellular confluence, are not relevant in vivo. However, a recent report of apparent adipocyte dedifferentiation in vivo upon application of a tissue expander in rats implicated mechanical signals in driving adipocyte plasticity^33^. Furthermore, adipocyte-to-myofibroblast fate switch during skin wound healing progresses beginning from the wound edge^5^, indicating that microenvironmental signals are driving this cell identity transition.

Using a screening approach to target several signal transduction pathways involved in mechanotransduction we identified Rho GTP-ase as a candidate mediator of PPARγ downregulation in adipocytes under replating conditions. The Rho pathway is known to mediate cellular responses to force^34^ and to affect intracellular cytoskeletal tension^35^. However, the role of Rho GTP-ase in regulating adipocyte state loss remains to be mechanically tested, both in vitro and in vivo.

In the future alternative approaches to standard 2D tissue culture, such as engineered 3D models of adipose tissue^36^, may provide the needed middle ground allowing efficient tracking of live cell state dynamics while preserving the mechanical properties and architecture of the tissue. The importance of stimulus convergence in driving adipocyte state loss, as described here, warrants further studies aimed at identifying the underlying plasticity-inducing stimuli and the molecular mechanisms of their interdependence, with the goal of devising new ways to prevent adipocyte state loss in fibrosis and other diseases caused by cell plasticity.

## Methods

### Animals

All animal studies were conducted in accordance with the guidelines and regulations approved by the Administrative Panel on Laboratory Animal Care at the Stanford University School of Medicine. Mice were purchased from Jackson Laboratory. mT/mG B6.129(Cg)-Gt(ROSA)26Sortm4(ACTB-tdTomato,-EGFP)Luo/J (cat. 007676) and nT/nG B6;129S6-Gt(ROSA)26Sortm1(CAG-tdTomato*,-EGFP*)Ees/J (cat. 023035) mice were bred to B6;FVB-Tg(Adipoq-cre)1Evdr/J mice (cat. 010803).

### Cell culture and differentiation

Stromal vascular fraction (SVF) was isolated from inguinal subcutaneous fat pads of 4–8-week old female and male mice using a previously published approach^16^. Fat pads were minced and digested in a solution of collagenase type D (Roche, 11088866001, 1 mg/ml) and Dispase II (Sigma-Aldrich, D4693, 1 mg/ml) in PBS with 1 mM CaCl_2_ for 40 min at 37 °C with shaking. The digest was passed through sterile nylon mesh and centrifuged at 300 RCF for 5 min. The SVF-containing pellet was resuspended in culture medium (DMEM with 10% FBS + 100 U/mL pen/strep) with 2.5 mg/ml amphotericin B for 2–4 h, which was then replaced. Cells were grown in the presence of 2.5 mg/ml amphotericin B for up to seven days before the start of differentiation protocol. To differentiate the SVF, cells were plated in 12-well cell culture plates at 120,000 cells per well (day -1). At day 0, cells were treated with 250 mM IBMX (Sigma-Aldrich), 1 mM dexamethasone (Sigma-Aldrich), 1.75 nM insulin (Sigma-Aldrich) and 500 nM rosiglitazone (Cayman Chemical) in culture medium. At day 2, cells were treated with 1.75 nM insulin and 500 nM rosiglitazone in culture medium, and at day 4 with 1.75 nM insulin in culture medium for two more days. Differentiated SVF cells were maintained in culture medium with 1.75 nM insulin afterwards.

OP9 cells were cultured in MEM-α media (Invitrogen) containing 100 units/mL Penicillin, 100 mg/ml Streptomycin, and 292 mg/ml L-glutamate. The base media also contained either 20% Fetal Bovine Serum (FBS) for cell expansion or 10% FBS for cell differentiation. To induce differentiation of OP9 cells, a standard DMI protocol was used: confluent cells were treated with 250 mM IBMX (Sigma-Aldrich), 1 mM dexamethasone (Sigma-Aldrich), and 1.75 nM insulin (Sigma-Aldrich) for 48 h, followed by 1.75 nM insulin for 48 h. Afterwards differentiated OP9 cells were maintained in base medium with 1.75 nM insulin.

Mouse TGF-β 1 recombinant protein was obtained from Affymetrix (#14-8342-62) and used at the concentration of 2 ng/ml unless stated otherwise. The following chemical inhibitors were used: Y27632 (Fisher Scientific, 10 µM), PND-1186 (VS-4718, Fisher Scientific, 1 µM), and Rho Inhibitor I C3 (Cytoskeleton, 0.5 µg/ml).

### Generation of SBE4:mScarlet-I-NLS reporter OP9 line and PPARγ overexpression vectors

For the cloning of fluorescent reporter of TGF-β transcriptional response (SBE4:mScarlet-I-NLS), Gibson Assembly Master Mix (New England Biolabs) was used according to manufacturer’s protocol. PiggyBac vector PB-CMV-MCS-EF1a-Puro (System Biosciences) was first modified to include blasticidin resistance gene instead of the puromycin one and linearized using SfiI and XbaI. Smad2/3 response element was amplified from SBE4-Luc construct, which was a gift from Bert Vogelstein (Addgene plasmid # 16495)^37^, and cloned upstream of mScarlet-I sequence^38^ with in-frame nuclear localization sequence (NLS). The sequence was verified using Sanger sequencing and the construct was introduced into mCitrine-PPARG H2B-mTurquoise OP9 cells by co-transfection with PiggyBac transposase vector, followed by selection with blasticidin (Thermo Fisher Scientific).

To create PPARγ overexpression vectors, the pEGFP-C2 plasmid (Clontech) was linearized with BamHI restriction enzyme. Human PPARG1 and PPARG2 sequences were amplified using pSV Sport PPARG1 (#8886) and pSV Sport PPARG2 (Addgene #8862) and introduced into the pEGFP-C2 backbone using Gibson Assembly Master Mix (New England Biolabs), according to manufacturer’s protocol, and the sequences were verified using Sanger sequencing. Cells were transfected with the plasmids using Lipofectamine 2000 (Thermo Fisher Scientific), according to manufacturer’s protocol. The PB_SBE4:mScarlet-I-NLS plasmid has been deposited with Addgene (#78241).

### siRNA-mediated gene silencing

*Pparg* siRNA and the non-targeting control siRNA were purchased from Dharmacon and transfected into OP9 cells using Lipofectamine RNAiMax (Invitrogen) according to the manufacturer’s protocol using 5 pmol siRNA per 96-well plate well.

### Immunofluorescent (IF) staining

All cultured cells were fixed with 4% PFA in PBS for 30 min at room temperature, followed by three washes with PBS. Cells were then permeabilized with 0.1% Triton X-100 in PBS for 15 min on ice, followed by blocking with 5% bovine serum albumin (BSA, Sigma Aldrich) in PBS. The cells were incubated with primary antibodies in 2% BSA in PBS overnight at 4 °C: mouse anti-PPARγ (Santa Cruz Biotech, sc-7273, 1:1,000), rabbit anti-CEBPα (Santa Cruz Biotech, sc-61, 1:1,000), chicken anti-GFP (Fisher Scientific, NB1001614, 1:1,000). After washing, cells were incubated with Hoechst (1:10,000) and secondary antibodies in 2% BSA / PBS overnight at 4 °C. Secondary antibodies included AlexaFluor-conjugated anti-rabbit and anti-mouse antibodies (1:1,000, Invitrogen) and anti-chicken AlexaFluor488 antibody (Thermo Fisher Scientific, A11039, 1:1,000). Cells were washed three times with PBS prior to imaging.

### Fluorescent imaging

Imaging was conducted using either an ImageXpress MicroXL (Molecular Devices, USA) or a 3i (Nikon) epifluorescent microscope with a 10X objective. Live fluorescent imaging was conducted at 37 °C with 5% CO_2_. A camera bin of 2 × 2 was used for live imaging and 1 × 1 was used for fixed imaging. Cells were plated in optically clear 96-well plates: plastic-bottom plates (Costar, #3904) for fixed imaging or glass-bottom µ-Plate (Ibidi, #89626) for live imaging. Living cells were imaged in FluoroBrite DMEM media (Invitrogen) with 10% FBS, 1% Penicillin/Streptomycin and insulin to reduce background fluorescence. Depending on the experiment, images were taken every 12–15 min in different fluorescent channels: CFP, YFP and/or RFP. Total light exposure time was kept less than 500 ms for each time point. Several non-overlapping sites in each well were imaged. Cell culture media were changed at least every 48 h.

### Imaging data processing

Data processing of fluorescent images was conducted in MATLAB R2016a (MathWorks). Unless stated otherwise, fluorescent imaging data were obtained by automated image segmentation, tracking and measurement using the MACKtrack package for MATLAB^39^.

Quantification of PPARγ- and C\EBPα-positive cells was based on quantification of mean fluorescence signal over nuclei. Cells were scored as PPARγ- and C/EBPα-positive if the marker expression level was above a preset cut-off determined by the bimodal expression at the earliest analyzed time point. GFP-positive cells were scored based on the mean value of GFP fluorescence signal measured over cell nucleus being above a preset cutoff determined by analysis of the distribution in the population.

For live imaging data of OP9 cells, CFP channel was used for nuclear segmentation and cell tracking. Obtained single-cell traces were filtered to remove cells absent at endpoint, traces with more than 10 empty frames and a fraction of traces with maximal changes of mCitrine intensity, quantified as the maximum of a moving integral of the squared difference between mCitrine intensity and local average over a window of double the length of the window used for cell tracking. The filtering for the changes of PPARγ intensity was according to a set cut-off for all conditions, and the cut-off was chosen so that only up to 2% of traces were removed in control.

If cells were binned according to their mCitrine-PPARG expression, cells were binned based on their mean nuclear mCitrine expression in the first frame of the experiment, with the exception of SBE4:mScarlet-I-NLS reporter cells, which were binned based on the mCitrine-PPARG expression in the last frame prior to addition of stimulus.

To quantify activity of the SBE4:mScarlet-I-NLS reporter, mScarlet-I signal was measured integrated over the whole nuclear area and recalculated as the change over the preceding frame. Median of single-cell Δ[Integrated mScarlet-I-NLS]/Δt traces was then smoothened using a moving average over time window equal to double the number of frames used for accurate single-cell tracking in MACKtrack. If a cell trajectory present at the beginning of the experiment (parent cell) split into more trajectories (daughter cells), the mScarlet-I signal values for the parent were calculated as the mean of daughter cell trajectories.

### Western blot analysis

Protein concentrations were determined using the Bradford assay (Thermo Fisher Scientific). Samples were subjected to SDS-PAGE in polyacrylamide gels (NuPAGE 4–12% Bis–Tris Protein Gels, Thermo Fisher Scientific) and transferred onto PVDF membranes (Thermo Fisher Scientific, #PI-88518) using NuPAGE Novex system (Thermo Fisher Scientific). Proteins were detected using primary antibodies against PPARγ (Santa Cruz Biotech, sc-7273, 1:1,000) and GFP (Abcam, ab111258, discontinued, 1:2,000), secondary anti-goat (Thermo Fisher Scientific, #31402, 1:15,000) and anti-mouse peroxidase-conjugated antibodies (Abcam, ab97046, 1:15,000), and the SuperSignal Femto ECL kit (Thermo Scientific) according to the manufacturer’s protocol. Equal protein loading was verified using HRP-conjugated α-β-actin antibody (Santa Cruz, sc-47778).

### Statistics

Unless specified otherwise, data are expressed as mean ± standard error of the mean (S.E.M.). *p* values < 0.05 were considered statistically significant. Analyses were performed using PRISM software v. 7.04.

## Supplementary information

Supplementary Information

## Data Availability

All datasets generated during this study are available from the corresponding author on request.
