# Supplementary material for: TGF-β is insufficient to induce adipocyte state loss without concurrent PPARγ downregulation
Source: Sci Rep. 2020 Aug 21;10:14084. doi: 10.1038/s41598-020-71100-z (PMC7442643; doi:10.1038/s41598-020-71100-z)
Supplement: Supplementary file 1 — Supplementary Information [file 41598_2020_71100_MOESM1_ESM.pdf]

Supplementary Information

TGF-β is insufficient to induce adipocyte state loss without concurrent PPARγ downregulation

Brooks Taylor, Arnav Shah, and Ewa Bielczyk-Maczynska

Supplementary Figure S1

Analysis of GFP co-expression with adipocyte markers in differentiating stromal vascular fraction (SVF) cells from *Adipoq:Cre mT/mG* and *Adipoq:Cre nT/nG* mice. (a) Representative fluorescent images of immunofluorescent staining against GFP and PPARγ of differentiating *Adipoq:Cre mT/mG* SVF cells. Enlarged boxed image from day 6 is shown. (b) Representative fluorescent images of differentiating SVF from a *Adipoq:Cre nT/nG* mouse at day 6 of differentiation. (c) Density plots showing co-expression of GFP and adipocyte markers PPARγ and C/EBPα in single SVF cells from *Adipoq:Cre nT/nG* and negative control (*nT/nG*) mice over six days of differentiation. Cut-off values used to identify the GFP-, PPARγ- and CEBPα-positive cells are marked by dashed lines.

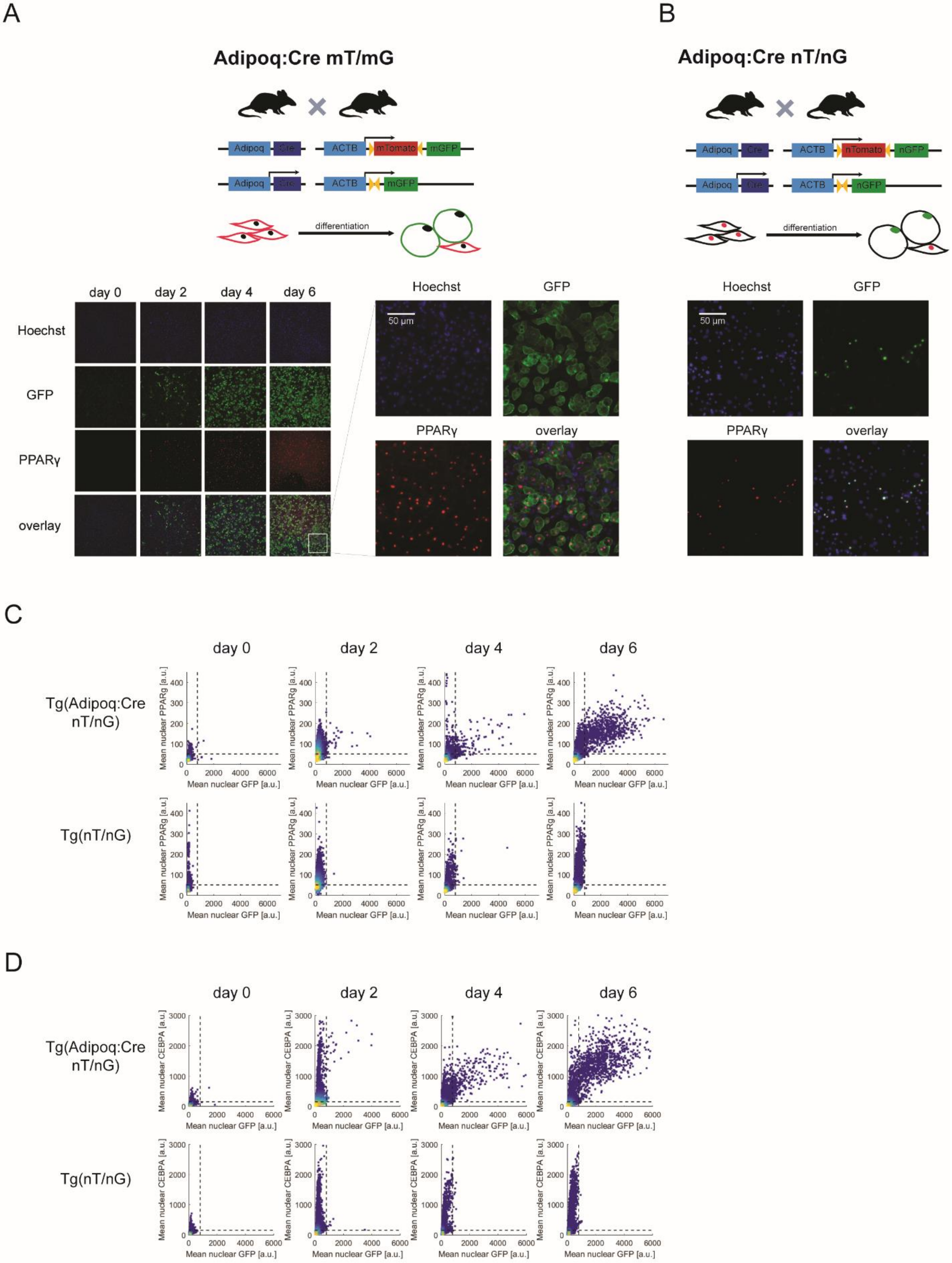
A
B
Adipoq:Cre mT/mG
Adipoq:Cre nT/nG
Adipoq
Cre
ACTB
mTomato
mGFP
nTomato
nGFP
differentiation
day 0
day 2
day 4
day 6
Hoechst
GFP
PPARγ
overlay
50 µm
C
D
Tg(Adipoq:Cre nT/nG)
Tg(nT/nG)
Mean nuclear PPARg [a.u.]
Mean nuclear CEBPA [a.u.]
Mean nuclear GFP [a.u.]

Supplementary Figure S2

Average number of all GFP-positive and all differentiated nonreplated *Adipoq:Cre mT/mG* cells, corresponding to Figure 1. n=6-15 technical replicates per data point.

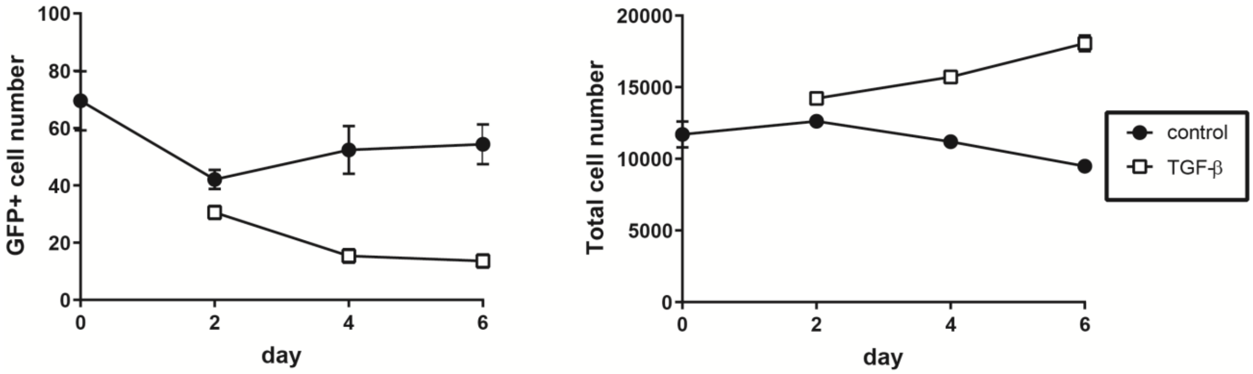

Supplementary Figure S3

Analysis of mCitrine-PPARG expression in differentiated nonreplated cells tracked for 36 h. TGF-β added at 2 h. Data corresponding to Figure 2.

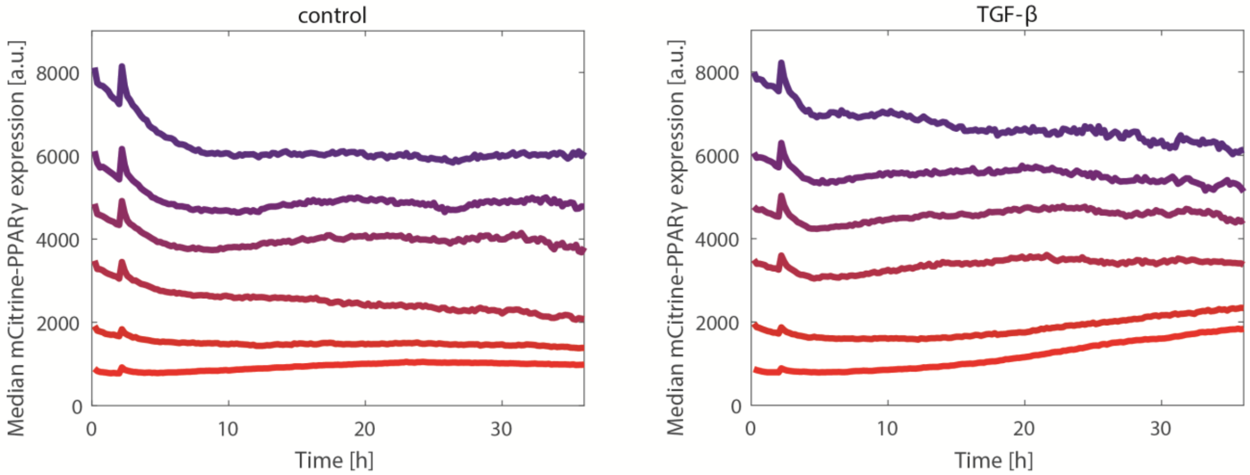

Supplementary Figure S4

TGF-β treatment leads to clump formation in OP9 but not SVF-derived primary cells. At the end of adipogenic differentiation protocol OP9 and SVF cells were either kept in control media or stimulated with TGF-β (2 ng/ml) for 96 h. Nuclei, which were counterstained with DAPI, are shown. A cell clump present in OP9 cells stimulated with TGF-β is indicated with the red arrow.

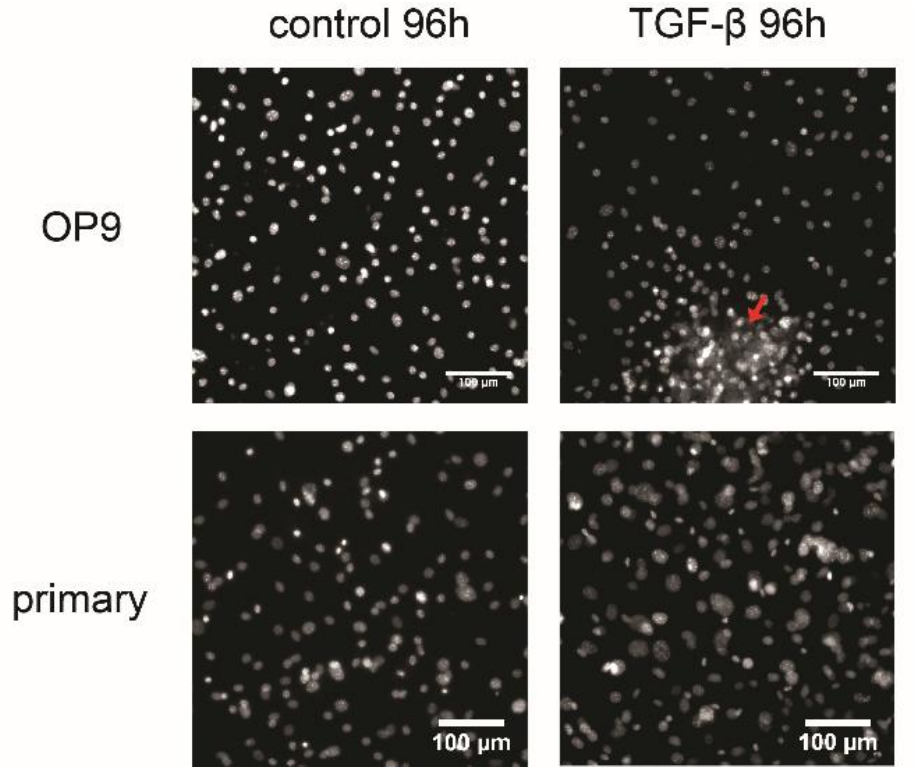

Supplementary Figure S5

Activation of TGF-β signaling in adipocytes temporally follows cell clump formation. (a-b) Differentiated mCitrine-PPARG SBE4:mScarlet-I-NLS OP9 cells were imaged live while treated with TGF-β beginning at 2 h. Results of one experiment representative for two independent experiments. (a) Binning of cells into six groups based on their mCitrine-PPARG expression in the last frame before stimulus addition (2h), shown for the control group. (b) Average number of neighbors, quantified as other nuclei present within 38 μm radius of the nucleus center for every cell. (c) Time course analysis of TGF-β-dependent transcriptional response depending on the initial mCitrine-PPARγ expression in mCitrine-low (bins 1-3) and mCitrine-high (bins 4-6) cells shows reporter upregulation, indicated by positive values of the change in integrated nuclear mScarlet-I signal over time (ΔmScarlet/Δt), in mCitrine-high cells. Median trace for each bin is shown. 46 h of treatment with TGF-β (2 ng/ml), or with basal media in control, started after two hours of pre-incubation with basal media. (b-c) Vertical line denotes the time point (~27 h) when SBE4:mScarlet-I-NLS reporter starts to indicate TGF-β signaling activation in mCitrine-high cells (bins 4-6).

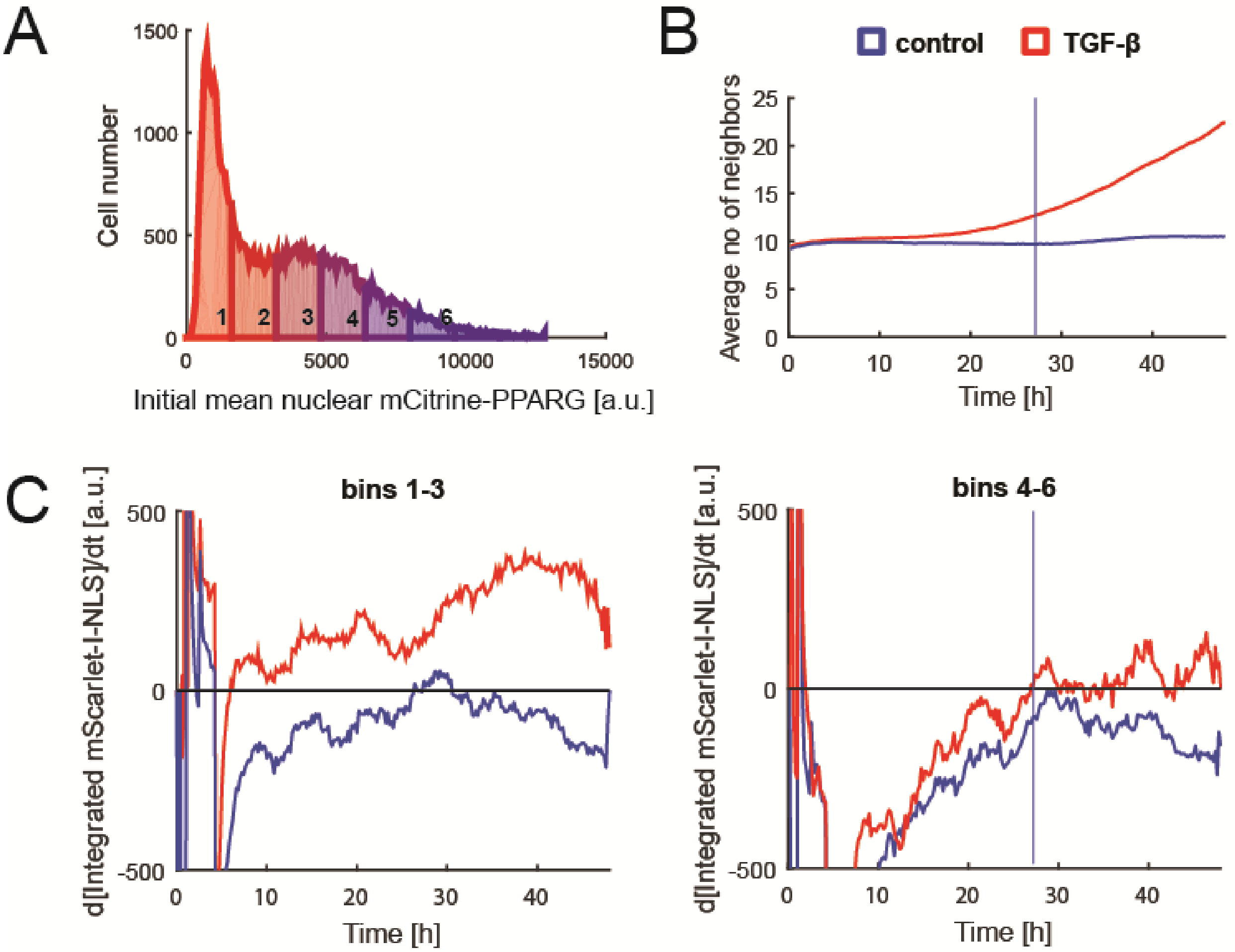

Supplementary Figure S6

Overexpression of PPARγ1 or PPARγ2 in undifferentiated subconfluent OP9 cells does not lead to upregulation of the endogenous PPARγ levels. Western blotting from transfected OP9 cells harvested 24 h after transfection against PPARγ and GFP. Predicted molecular weight of endogenous and overexpressed proteins is indicated.

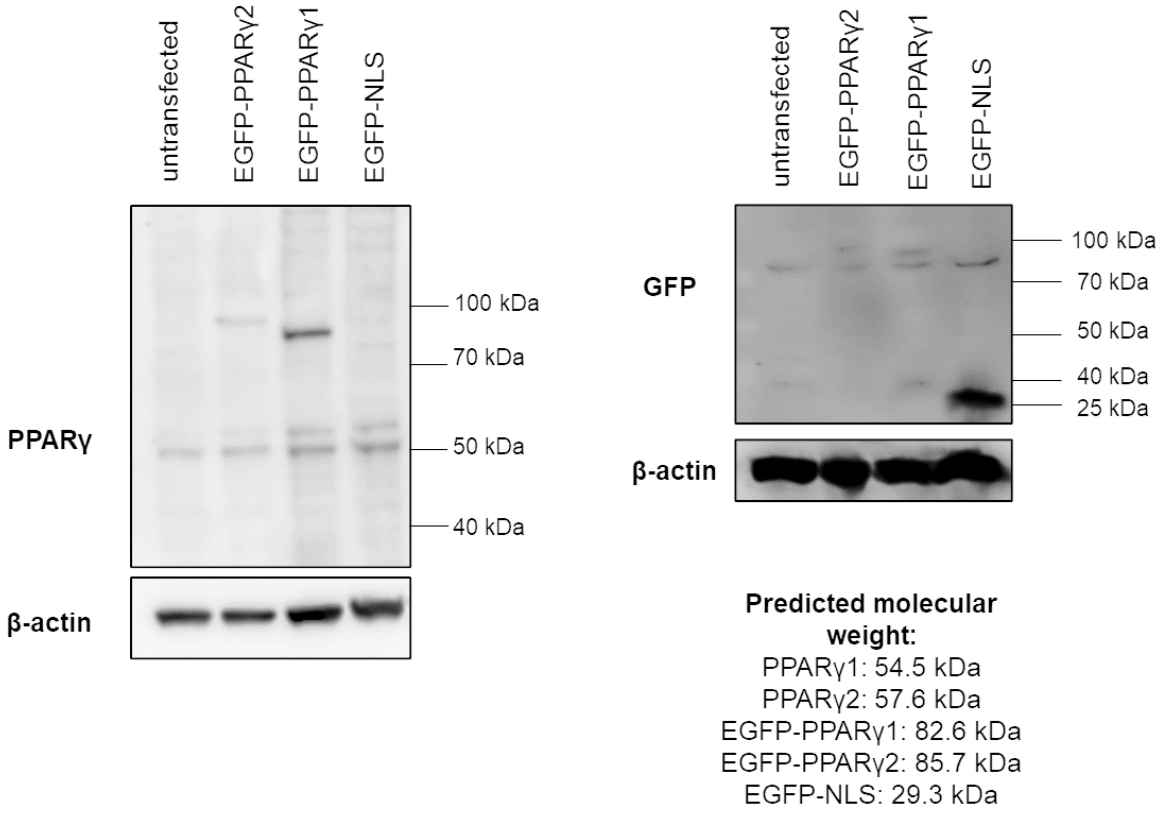

Supplementary Figure S7

Rho signaling may be involved in mediating Pparγ downregulation in adipocytes following replating. (a) Workflow used to identify pathways involved in Pparγ downregulation in adipocytes following replating. Initial mCitrine expression was used to identify differentiated cells (mCitrine-high cells, blue). The mean mCitrine expression in these cells was compared at 0, 12 and 24 h after replating. Stimuli were added immediately after replating. (b) Replated, differentiated mCitrine-PPARG OP9 cells were treated with chemical inhibitors of FAK (PND-1186), ROCK (Y27632), or Rho (C3 Rho inhibitor I) to test for their effect on mCitrine downregulation. Average and S.E.M. are shown, n=4 technical replicates, >346 cells per replicate. One-way ANOVA with Sidak correction; **, p<0.01; ***, p<0.001; n.s. – not significant. Results of one experiment representative of 2-3 independent experiments per inhibitor.

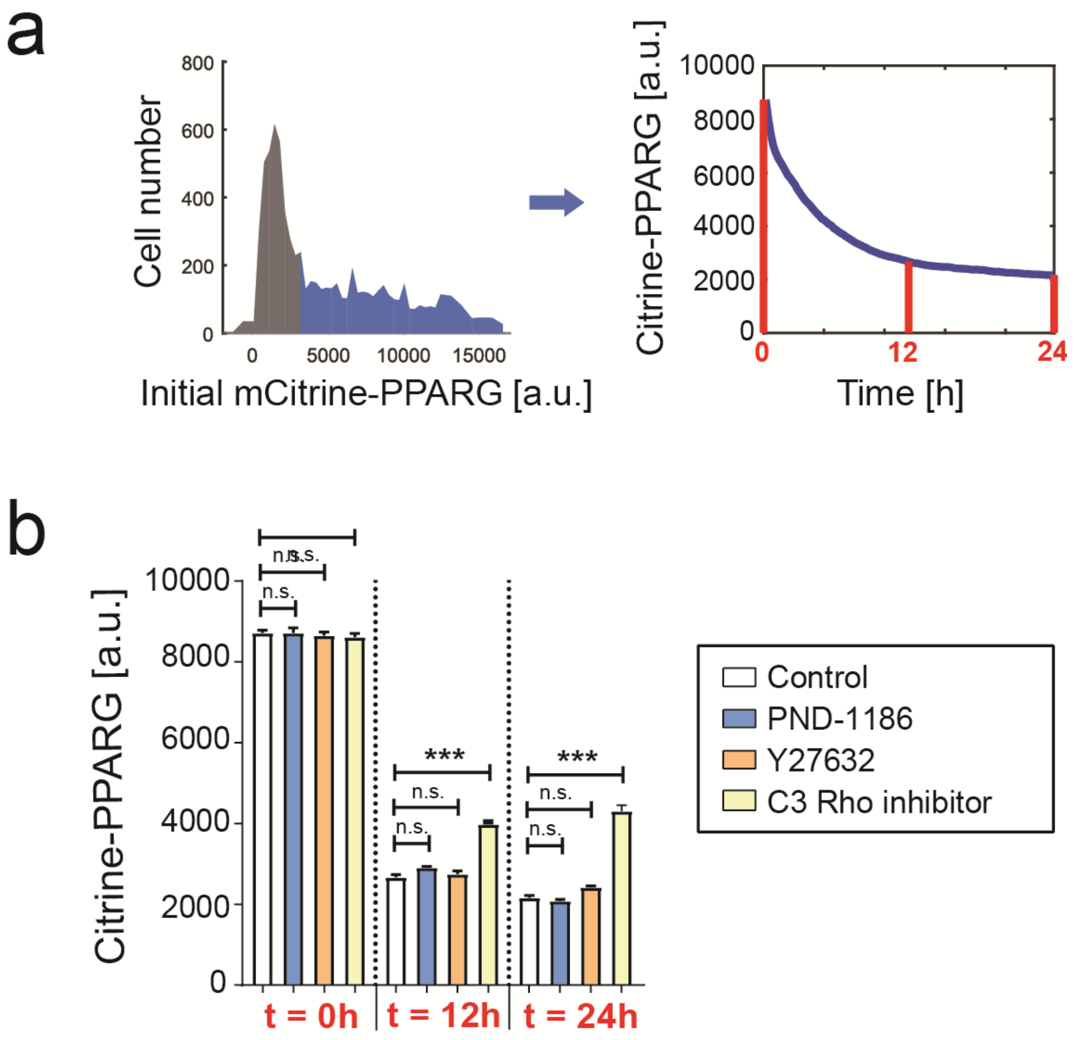

Supplementary Table S1

Number of cells analyzed, corresponding to Fig. 7b.

Plasmid	Experimental group	EGFP expression	Cell number
EGFP-PPARG2	Control	EGFP-	11,477
EGFP-PPARG2	Control	EGFP+	120
EGFP-PPARG2	TGF-β	EGFP-	11,925
EGFP-PPARG2	TGF-β	EGFP+	115
EGFP-PPARG1	Control	EGFP-	5,131
EGFP-PPARG1	Control	EGFP+	430
EGFP-PPARG1	TGF-β	EGFP-	5,978
EGFP-PPARG1	TGF-β	EGFP+	447
EGFP-NLS	Control	EGFP-	1,377
EGFP-NLS	Control	EGFP+	2,481
EGFP-NLS	TGF-β	EGFP-	1,046
EGFP-NLS	TGF-β	EGFP+	1,522
